# Activity Analysis of the P2X Receptor Antagonist PPADS against Signal Transducer and Activator of Transcription Proteins

**DOI:** 10.1002/cbic.202500454

**Published:** 2025-07-24

**Authors:** Angela Berg, Martin Gräber, Thorsten Berg

**Affiliations:** ^1^ Institute of Organic Chemistry Leipzig University Johannisallee 29 04103 Leipzig Germany; ^2^ Department of Molecular Biology Max Planck Institute of Biochemistry, and Center for Integrated Protein Science Munich (CIPSM) Am Klopferspitz 18 82152 Martinsried Germany

**Keywords:** biological activity, inhibitors, protein–protein interactions, Src homology 2 domains, transcription factors

## Abstract

The small molecule PPADS is a P2X receptor antagonist. Analysis of a library of known bioactive compounds in competitive fluorescence polarization‐based assays indicates that PPADS and its isomer iso‐PPADS also possess activity against members of the signal transducer and activator of transcription (STAT) family of transcription factors, in particular STAT4, STAT5a, and STAT5b. These results are also supported by HTRF assays. PPADS protects STAT4 and STAT5a/STAT5b from thermal degradation in cell lysates to a higher extent than STAT3. Combined data from this article and a previous article suggest that aromatic disulfonate antagonists of P2X receptors might serve as promising starting points for the development of inhibitors of STAT proteins.

## Graphical abstract

The P2X receptor antagonist PPADS possesses activity against members of the signal transducer and activator of transcription (STAT) family of transcription factors, in particular STAT4, STAT5a, and STAT5b. PPADS protects STAT4 and STAT5a/STAT5b from thermal degradation in cell lysates to a higher extent than STAT3. The data suggest that aromatic disulfonate antagonists of P2X receptors may represent promising starting points for the development of inhibitors of STAT proteins.

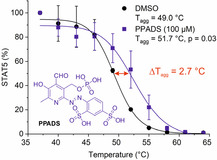

## 1 Introduction

Signal transducers and activators of transcription (STATs) are latent cytoplasmic transcription factors that convey signals from the cell surface to the nucleus.^[^ ^1^ ^]^ All members of the STAT family contain a Src homology 2 (SH2) protein–protein interaction domain, which mediates binding to activated cell surface receptors, growth factor receptors, and nonreceptor tyrosine kinases. The key recognition element of SH2 domains is phenyl phosphate, as part of the side chain of an O‐phosphorylated tyrosine residue. We have previously developed selective inhibitors of STAT5a,^[^ ^2^, ^3^ ^]^ STAT5b,^[^ ^4^, ^5^, ^6^, ^7^ ^]^ and STAT5a/5b^[^ ^8^ ^]^ by adding specific recognition elements to phenyl phosphate. Overactivation of STAT5b in particular is associated with tumorigenesis^[^ ^9^ ^]^ and has been found in many human cancers.^[^ ^10^ ^]^ We also recently presented biaryl phosphates and phosphonates as the first selective inhibitors of STAT4,^[^ ^11^ ^]^ which has been implicated in the pathogenesis of autoimmune diseases, such as multiple sclerosis, rheumatoid arthritis, and type 1 diabetes, and is a candidate therapeutic target.^[^ ^12^ ^]^ The design approach of tailoring substituents onto phenyl phosphate to obtain selective inhibitors of individual STAT proteins was based on our discovery that 2‐carboxyphenyl phosphate (fosfosal) inhibits the STAT5b SH2 domain with specificity over the SH2 domains of other STAT proteins.^[^ ^13^, ^14^ ^]^ Further known bioactive compounds for which activity against STAT5 proteins could be demonstrated include the P2X receptor antagonists suramin, NF023, and NF449.^[^ ^15^ ^]^

## 2 Results and Discussion

**Figure 1:**
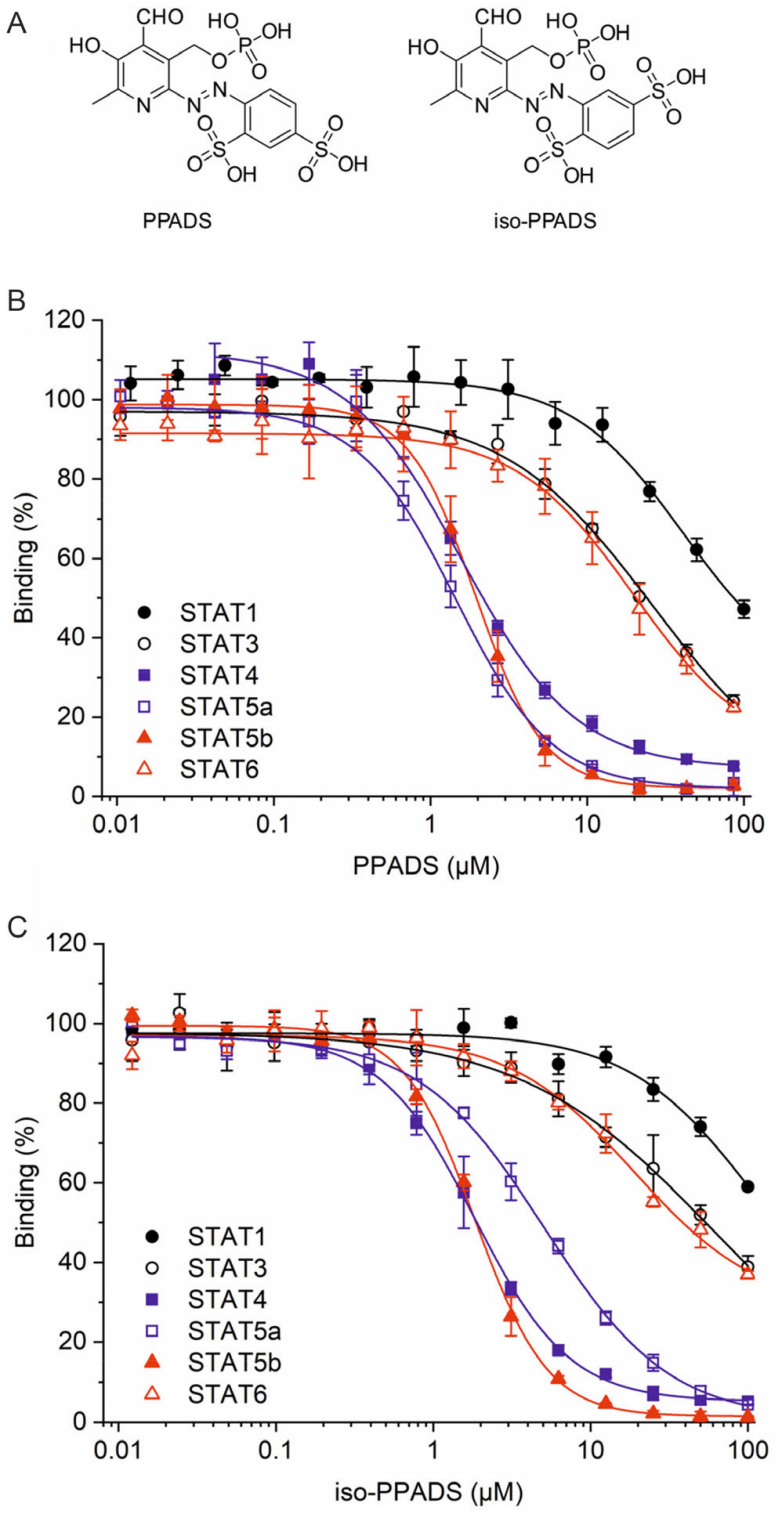
A) Structures of PPADS and iso‐PPADS. Activity of B) PPADS and C) iso‐PPADS against STAT proteins in FP assays in the absence of reducing agent after 1 h. Error bars represent standard deviations (*n* = 3).

Here, we report that screening of a chemical library consisting of ≈5000 known bioactive compounds^[^ ^13^ ^]^ using fluorescence polarization (FP)‐based competitive assays^[^ ^13^, ^15^, ^16^, ^17^ ^]^ identified the P2X receptor antagonist PPADS (**1**)^[^ ^18^ ^]^ (pyridoxalphosphate‐6‐azophenyl‐2',4'‐disulfonic acid, **Figure** **1**A) as an inhibitor of multiple STAT proteins. Dose‐dependent activity analysis revealed that PPADS inhibits STAT4 (IC_50_ = 2.2 ± 0.2 μM), STAT5a (IC_50_  = 1.5 ± 0.2 μM), and STAT5b (IC_50_ = 2.0 ± 0.3 μM) with similar activities (Figure 1B). In contrast, STAT1 (IC_50_ = 87.4 ± 6.6 μM), STAT3 (IC_50_ = 22.6 ± 1.6 μM), and STAT6 (IC_50_ = 20.1 ± 4.6 μM) were inhibited to a significantly lesser extent. Inhibition of STAT4 by PPADS did not increase with longer incubation periods (Supporting Information Figure S1A, Table S1), arguing against the possibility that the aldehyde functionality of PPADS engages in Schiff base formation with a lysine residue in the respective STAT proteins. However, in the presence of the nucleophilic reducing agent DTT, inhibition decreased with time (Figure S1B and Table S1, Supporting Information), which might be caused by reduction of the azo group, as has previously been reported for other compounds bearing a diarylazo moiety.^[^ ^19^, ^20^ ^]^ In the presence of the nonnucleophilic reducing agent TCEP, STAT4 inhibition was stable over time (Figure S1C and Table S1, Supporting Information).

Iso‐PPADS (**2**, Figure 1A), a regioisomer of PPADS in which one of the sulfonate groups is placed in the *meta*‐instead of the *para*‐position, displays antagonistic activity against P2X receptors in a similar manner to PPADS.^[^ ^21^ ^]^ The activity of iso‐PPADS against STAT4 (IC_50_  = 1.9 ± 0.2 μM, Figure 1C) and STAT5b (IC_50_  = 1.9 ± 0.1 μM) in FP assays was comparable to the activity of PPADS. Iso‐PPADS was two‐ to three‐fold less active than PPADS against STAT1 (41 ± 0.4% inhibition at 100 μM, the highest concentration tested), STAT3 (IC_50_  = 54 ± 14 μM), STAT5a (IC_50_  = 4.8 ± 0.4 μM), and STAT6 (IC_50_  = 66 ± 12 μM).

**Figure 2:**
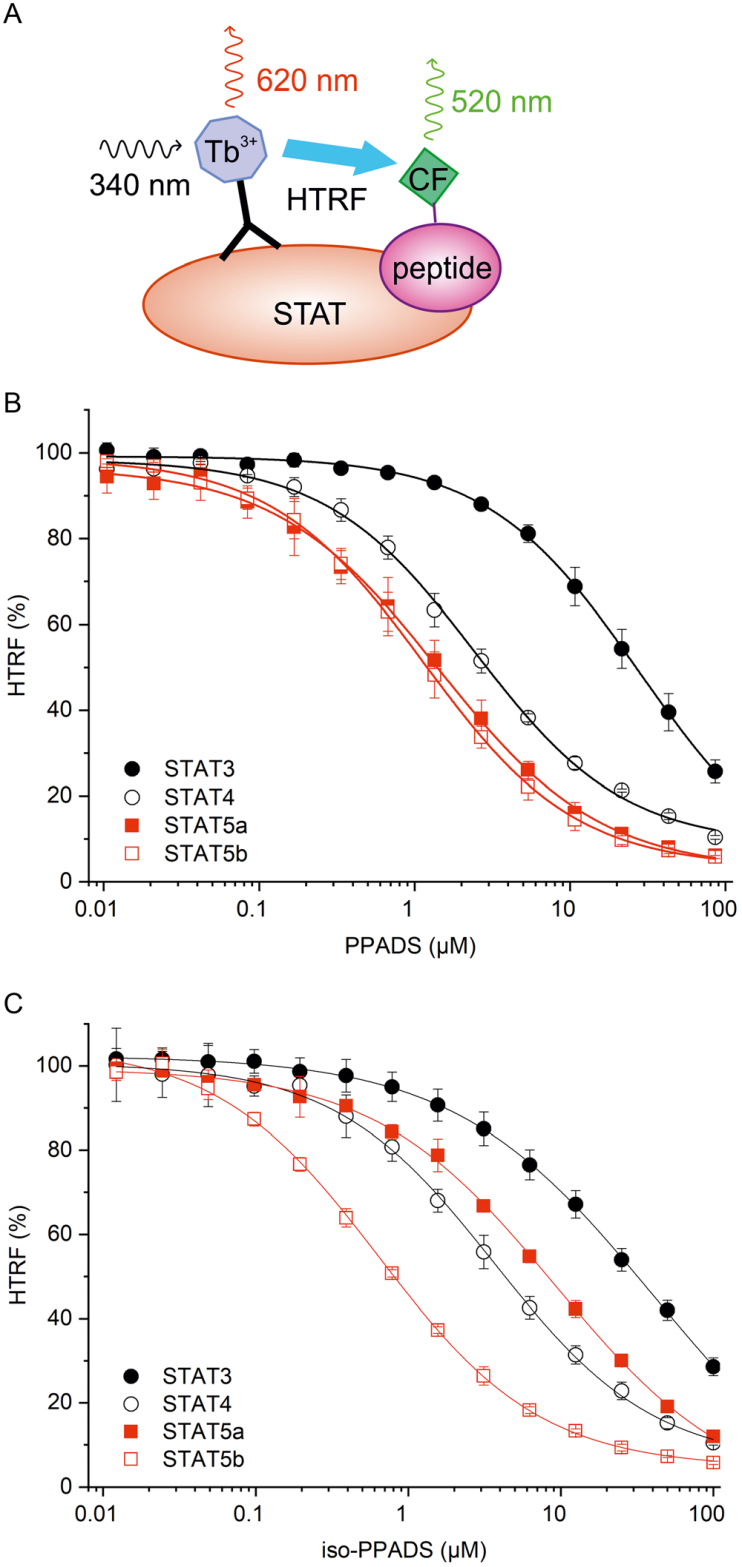
A) Principle of the HTRF assay. Activity of B) PPADS and C) iso‐PPADS against selected STAT proteins in HTRF assays. The HTRF ratio (see Supporting Information for details of the calculations) in the absence of inhibitor is defined as 100% HTRF. Error bars represent standard deviations (*n* = 3).

The azo dyes PPADS and iso‐PPADS have a deep red color in aqueous solution. Although the total intensity readings in FP assays did not increase in the presence of either compound, we wanted to verify that the apparent activity was not influenced by autofluorescence of the test compounds. In order to exclude this possibility, we established homogeneous time‐resolved fluorescence resonance energy transfer (HTRF) assays to confirm the degree of binding between STAT4, STAT5a, and STAT5b and the respective 5‐carboxyfluorescein‐labeled binding peptides used in the FP assay. In addition, an HTRF assay was established for STAT3, one of the STAT proteins targeted to a lesser extent in FP assays, as a specificity control (Figure 1B,C). The STAT protein constructs each contain a 6x‐His‐tag.^[^ ^15^, ^16^, ^17^, ^22^ ^]^ A Tb^3+^‐cryptate fused to an anti‐6x‐His antibody was chosen as the long‐lived fluorescent donor, while the carboxyfluorescein linked to the respective STAT‐binding peptides served as the acceptor (**Figure** **2**A). Emission was measured with a time delay of 60 μs after excitation, allowing any initial compound fluorescence to decay beforehand. Binding peptides lacking the fluorophore were used as positive controls to verify competitive inhibition (Figure S2 and Table S2, Supporting Information). The activities of PPADS in HTRF assays against STAT4 (IC_50_ = 2.92 ± 0.36 μM), STAT5a (IC_50_  = 1.41 ± 0.35 μM), and STAT5b (IC_50_  = 1.22 ± 0.27 μM) were similar to those determined in FP assays (Figure 2B). STAT3 was inhibited to a tenfold lesser extent than the other STAT proteins in the HTRF assay (IC_50_  = 29.6 ± 4.0 μM), which also reflects the FP assay results (Figure 1C). For iso‐PPADS, the activities in HTRF assays against STAT4 (IC_50_ = 8.6 ± 1.3 μM, Figure 2C), STAT5a (IC_50_  = 8.2 ± 0.7 μM), STAT5b (IC_50_  = 0.8 ± 0.1 μM), and STAT3 (IC_50_  = 31.9 ± 4.0 μM) were comparable to those determined in FP assays (Figure 1C), supporting the notion that the observed inhibition does not result from compound autofluorescence. To our knowledge, these assays represent the first application of HTRF assays for the activity analysis of small molecules acting on the SH2 domain of STAT proteins.

Although the addition of a Tb^3+^‐labeled antibody to the components of a carboxyfluorescein‐based FP assay is an effective approach by which to establish an HTRF assay (Figure 2A), it is possible that this setup might overestimate the activity of compounds able to absorb light in the green range of the spectrum (≈500–570 nm), which PPADS does to a small extent (Supporting Information Figure S3). The emission of the acceptor carboxyfluorescein is measured at 520 nm in the HTRF assay, with the emission of the terbium cryptate donor luminescence measured using an emission peak at 620 nm. The ratio of emissions at 520/620 nm represents the measurement of energy transfer efficiency. It is, therefore, theoretically possible for a compound that preferentially absorbs light at 520 nm over 620 nm to create an artificially reduced read‐out of energy transfer efficiency by filtering out the acceptor emission from the solution before it reaches the detector. However, since the profiles of selectivity of PPADS and iso‐PPADS in both HTRF and FP assays remain similar, including strongest inhibition of STAT5b and reduced activity against STAT3, it is unlikely that absorbance of the acceptor emission by the dyes alters the read‐out in HTRF assays in a meaningful way. As quantification of HTRF is determined by ratiometric analysis of donor and acceptor emissions, any absorption of incident light at the excitation wavelength of 340 nm by PPADS (Supporting Information Figure S3) should not affect the HTRF ratio.

**Figure 3:**
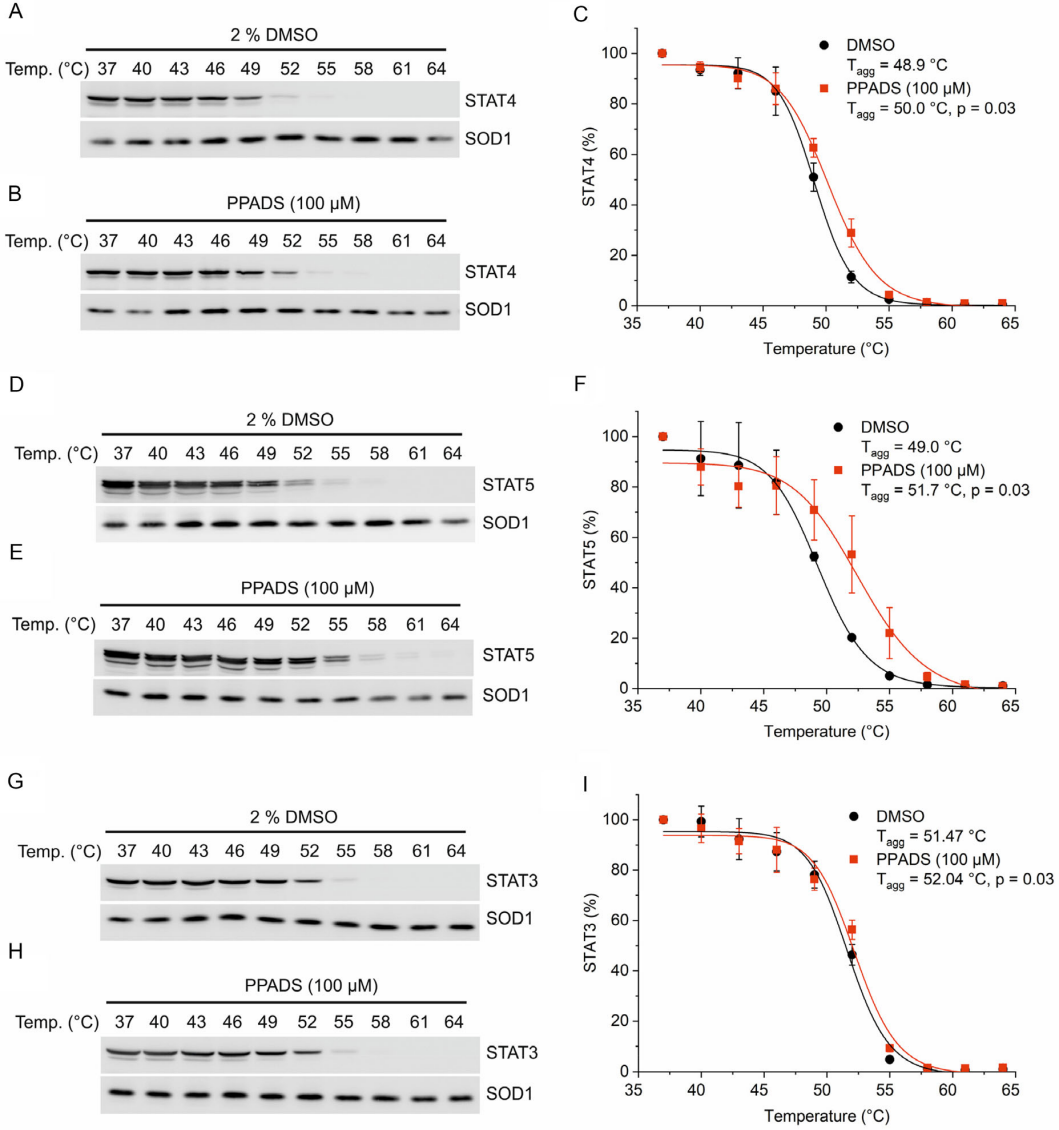
Thermally induced aggregation of STAT proteins is inhibited in the presence of 100 μM PPADS. A) STAT4 in lysates from NK‐92 cells in the presence of the DMSO control and B) PPADS, with C) quantitation of the STAT4 protein bands from triplicate experiments. D) STAT5 in lysates from K‐562 cells in the presence of DMSO and E) PPADS, with F) quantitation of the STAT5 protein bands from triplicate experiments. G) STAT3 in lysates from NK‐92 cells in the presence of DMSO and H) PPADS, with I) quantitation of the STAT3 protein bands from triplicate experiments. Error bars represent standard deviations (*n* = 3). *p*‐values represent the results from Student's *t*‐test, two‐tailed, two‐sample equal variance. Uncropped blot pictures are shown in Figure S5, Supporting Information.

Target inhibition in biochemical assays using purified proteins does not necessarily mean that the same target will be engaged in the context of a cellular environment. One method by which binding of small molecules to their expected targets in cell lysates can be inferred is the cellular thermal shift assay (CETSA).^[^ ^23^, ^24^ ^]^ This assay is based on the observation that reversible protein binding by a small molecule typically stabilizes the bound protein against thermal denaturation. In total cell lysates from the STAT4‐expressing natural killer cell line NK‐92, the aggregation temperature of STAT4 (*T*_agg_ = 48.9 ± 0.4 °C, **Figure** **3**A) was increased to 50.0 ± 0.4 °C in the presence of 100 μM PPADS, representing an increase of the aggregation temperature by 1.1 °C (*p* = 0.03, Figure 3B,C). This is a higher degree of stabilization than was observed in the presence of the high‐affinity STAT4‐binding peptide Ac‐GpYLPQNID at 100 μM (Δ*T*_agg_ = 0.5 °C, *p* = 0.08).^[^ ^11^ ^]^ The aggregation temperature of total STAT5 (STAT5a and STAT5b, *T*_agg_ = 49.0 °C, Figure 3D) in lysates from K562 cells, a frequently used cell line for assessing the activity of STAT5a/5b inhibitors,^[^ ^6^ ^]^ was also increased by 100 μM PPADS (*T*_agg_ = 51.7 °C, Δ*T*_agg_ = 2.7 °C, *p* = 0.03, Figure 3D,E). This is a larger shift in aggregation temperature than was observed in the presence of the positive control peptide DTpYLVLDKWL at 100 μM (*T*_agg_ = 49.5 °C, Δ*T*_agg_ = 0.5 °C, *p* = 0.57, Supporting Information Figure S4) and even at 500 μM (*T*_agg_ = 50.6 °C, Δ*T*_agg_ = 1.6 °C, *p* = 0.02, Supporting Information Figure S4).

STAT3 was again chosen as a specificity control for CETSA analysis, because it had already been used as control in HTRF assays (Figure 2B), and because we had previously developed CETSA for STAT3 in NK‐92 lysates.^[^ ^11^ ^]^ The aggregation temperature of STAT3 in total cell lysates from NK‐92 cells (*T*_agg_ = 51.47 °C, Figure 3G) was increased by only 0.57 °C (*T*_agg_ = 52.04 °C, *p* = 0.03, Figure 3H,I). While the statistical significance of this temperature shift is equal to those observed for STAT4 and STAT5, we previously reported that the positive STAT3 control peptide Ac‐pYLPQTV‐NH_2_ mediates a much stronger thermal shift (Δ*T*_agg_ = 1.9 °C, *p* = 0.0002) at the same concentration of 100 μM.^[^ ^11^ ^]^ This suggests that thermal stabilization of STAT3 is easier to obtain than that of STAT4 and STAT5. The relatively low thermal shifts obtained even with the high‐affinity control peptides are consistent with the fact that the SH2 domain of STATs only comprises a small proportion (≈100 amino acids) of the endogenous full‐length proteins (between 748 and 793 amino acids for STAT3/4/5a/5b).^[^ ^25^ ^]^ This limits the extent by which a STAT SH2 domain ligand can protect the protein against thermally induced protein motions which lead to misfolding, followed by aggregation and precipitation. The CETSA experiments were carried out in cell lysates, not in living cells, to account for the putative inability of PPADS and iso‐PPADS to cross the cell membrane owing to their large number of negative charges.

## 3 Conclusion

PPADS and iso‐PPADS are aromatic disulfonate antagonists of P2X receptors,^[^ ^26^ ^]^ as are suramin, NF023, and NF449, for which we previously reported in vitro activity against STAT5a and STAT5b.^[^ ^15^ ^]^ While further efforts are needed to investigate the interaction of PPADS with STAT proteins in molecular detail and to explore the specificity of PPADS against protein–protein interaction domains on a wider scale, we conclude that aromatic disulfonates may have an intrinsic propensity to target the SH2 domains of STAT proteins and may represent promising starting points for the development of STAT protein inhibitors. Further experiments would be necessary to determine the cell permeability of PPADS and iso‐PPADS, with subsequent development of derivatives with a reduced number of negative charges and/or masking those charges as appropriate. In addition, the azo moiety could be replaced by more stable chemical entities. Chemical agents improved by these approaches could furthermore serve as the basis for the development of proteolysis‐targeting chimeras^[^ ^27^ ^]^ or molecular glues^[^ ^28^ ^]^ against STAT proteins.

## Conflict of Interest

The authors declare no conflict of interest.

## Supporting information

Supplementary Material

## Data Availability

The data that support the findings of this study are available in the supplementary material of this article.
