# Supplementary material for: Activity Analysis of the P2X Receptor Antagonist PPADS against Signal Transducer and Activator of Transcription Proteins
Source: Chembiochem. 2025 Jul 24;26(17):e202500454. doi: 10.1002/cbic.202500454 (PMC12442245; doi:10.1002/cbic.202500454)
Supplement: Supplementary file 1 — Supplementary Material [file CBIC-26-e202500454-s001.pdf]

Supporting Information

Activity Analysis of the P2X Receptor Antagonist PPADS against Signal Transducer and Activator of Transcription Proteins

Angela Berg,[a] Martin Gräber,[a,b] and Thorsten Berg*[a]

[a] Dr. A. Berg, Dr. M. Gräber, Prof. Dr. T. Berg
Institute of Organic Chemistry
Leipzig University
Johannisallee 29, 04103 Leipzig, Germany
E-mail: tberg@uni-leipzig.de

[b] Dr. M. Gräber
Department of Molecular Biology, Max Planck Institute of Biochemistry, and Center for Integrated Protein Science Munich (CIPSM)
Am Klopferspitz 18, 82152 Martinsried, Germany

Table of Contents

Figure S1 3
Figure S2 4
Figure S3 4
Figure S4 5
Figure S5 6
Figure S6 7
Table S1 7
Table S2 7
Screening of chemical libraries 8
Fluorescence polarization assays 8
HTRF assays 8
Cell culture and lysates 9
CETSA 9
Western blotting 9
Supporting references 10

Figure S1

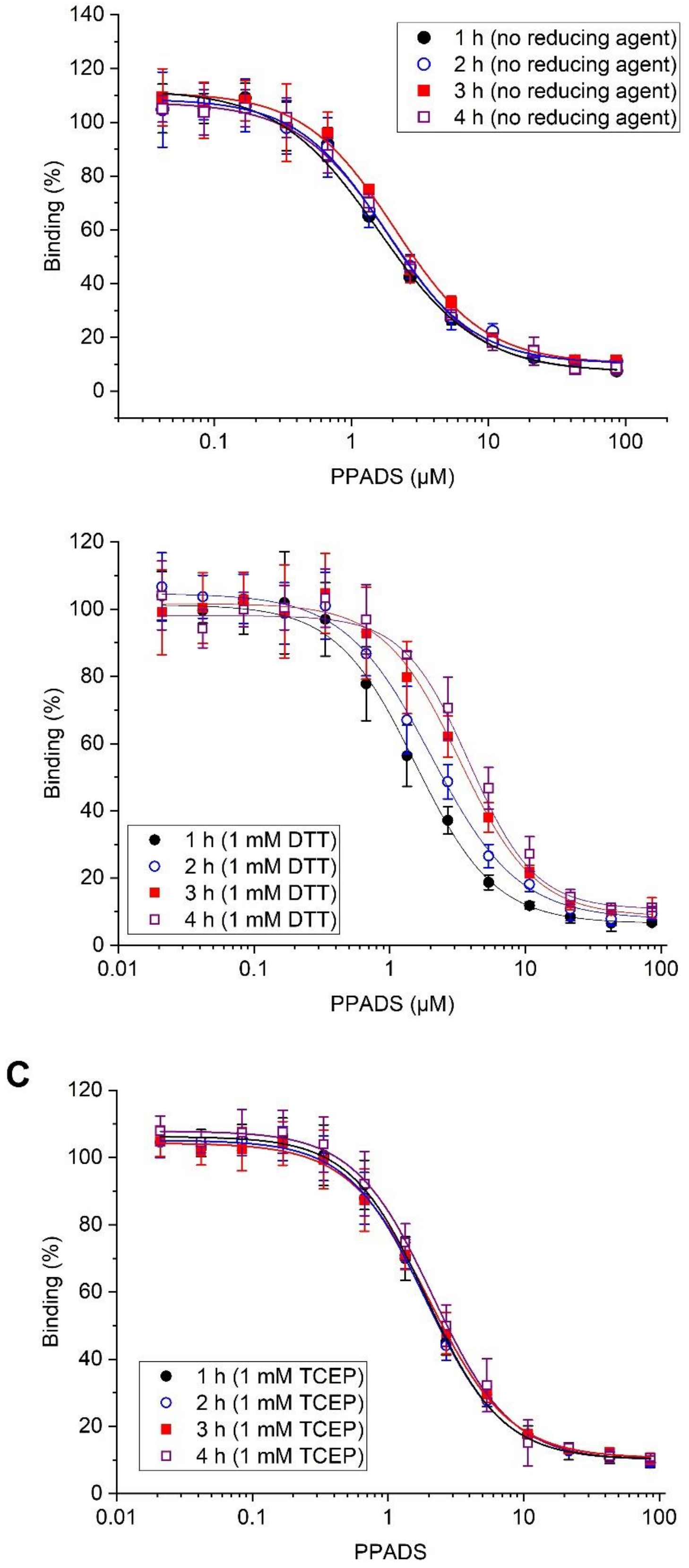

Figure S1: Stability of inhibition of STAT4 by PPADS over time in FP assays in the presence of A) no reducing agent, B) 1 mM DTT or C) 1 mM TCEP. Error bars represent standard deviations (n = 3).

Figure S2

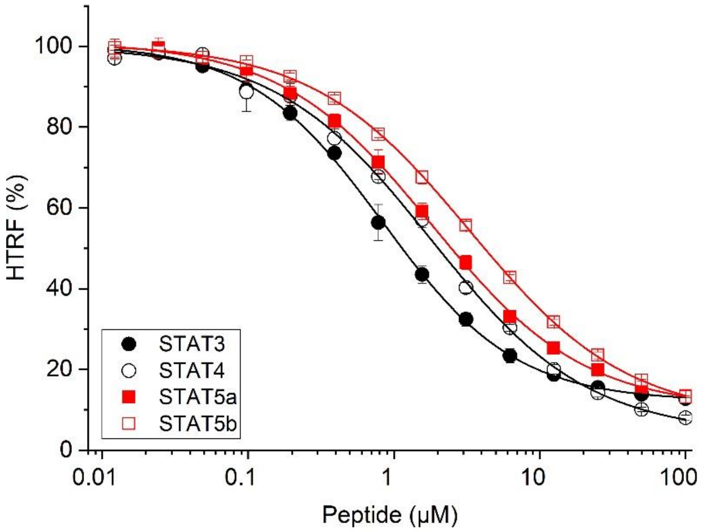

Figure S2: Activity of positive control peptides against the respective STAT proteins in HTRF assays. Carboxyfluorescein (CF)-labeled sequences are STAT3: 5-CF-GpYLPQTV-NH_2, STAT4: 5-CF-GpYLPQNID, STAT5a/STAT5b: 5-CF-GpYLVLDKWL; unlabeled peptide sequences are STAT3: Ac-pYLPQTV-NH_2, STAT4: Ac-GpYLPQNID, STAT5a/STAT5b: DTpYLVLDKWL. The HTRF ratio in the absence of inhibitor is defined as 100 % HTRF. Error bars represent standard deviations (n = 3).

Figure S3

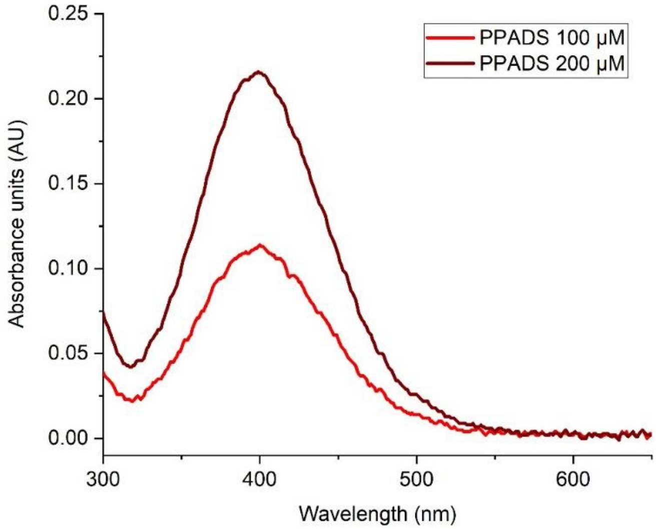

Figure S3: UV/Vis spectrum of PPADS in water at the indicated concentrations.

Figure S4

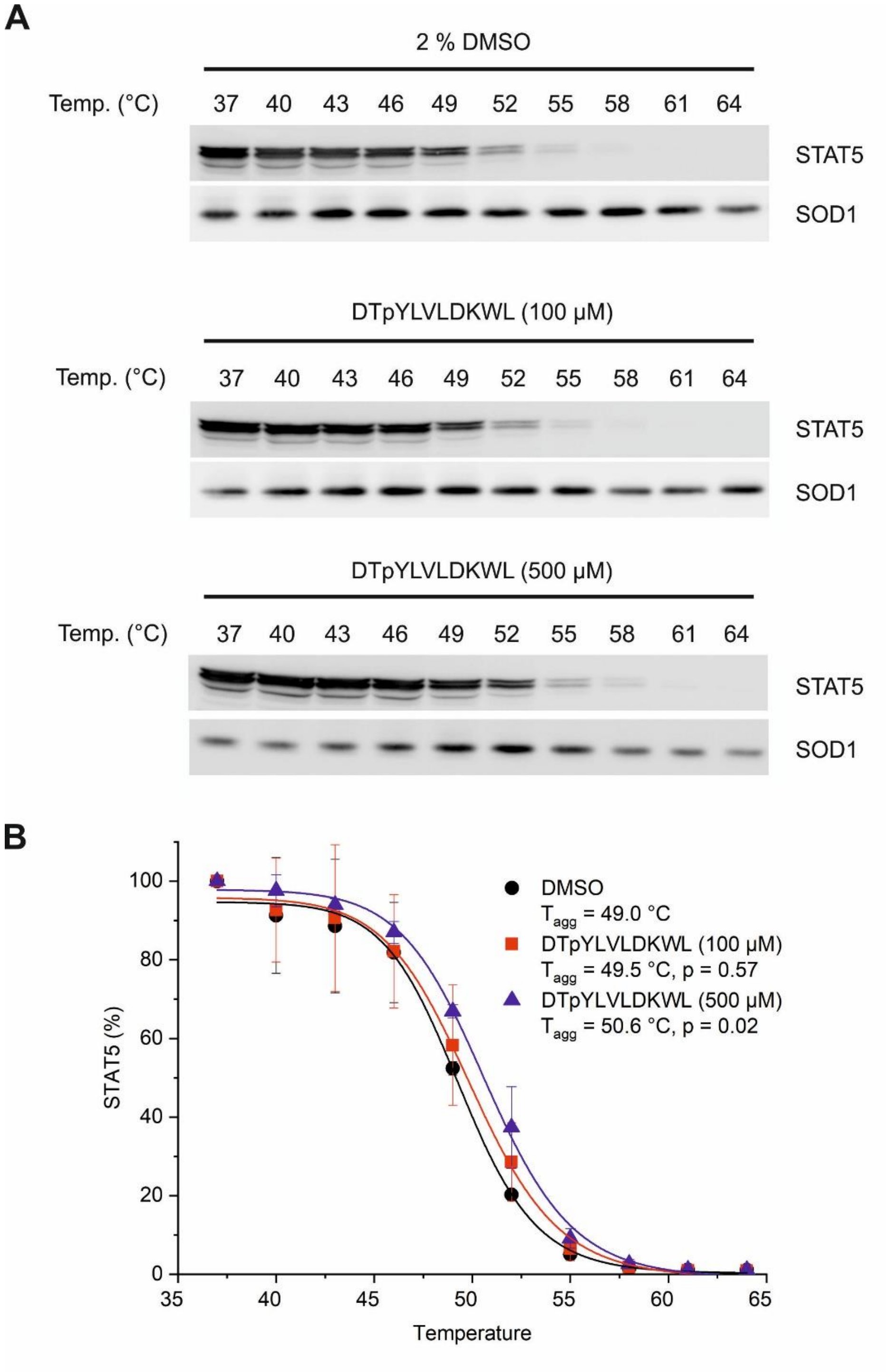

Figure S4: A) Thermal degradation of STAT5 from lysates of K562 cells in the presence of DMSO, 100 µM or 500 µM of the positive control peptide DTpYLVLDKWL, with B) quantitation of triplicate experiments. Error bars represent standard deviations (n = 3). p-values represent the results from Student's t-test, two-tailed, two-sample equal variance.

Figure S5

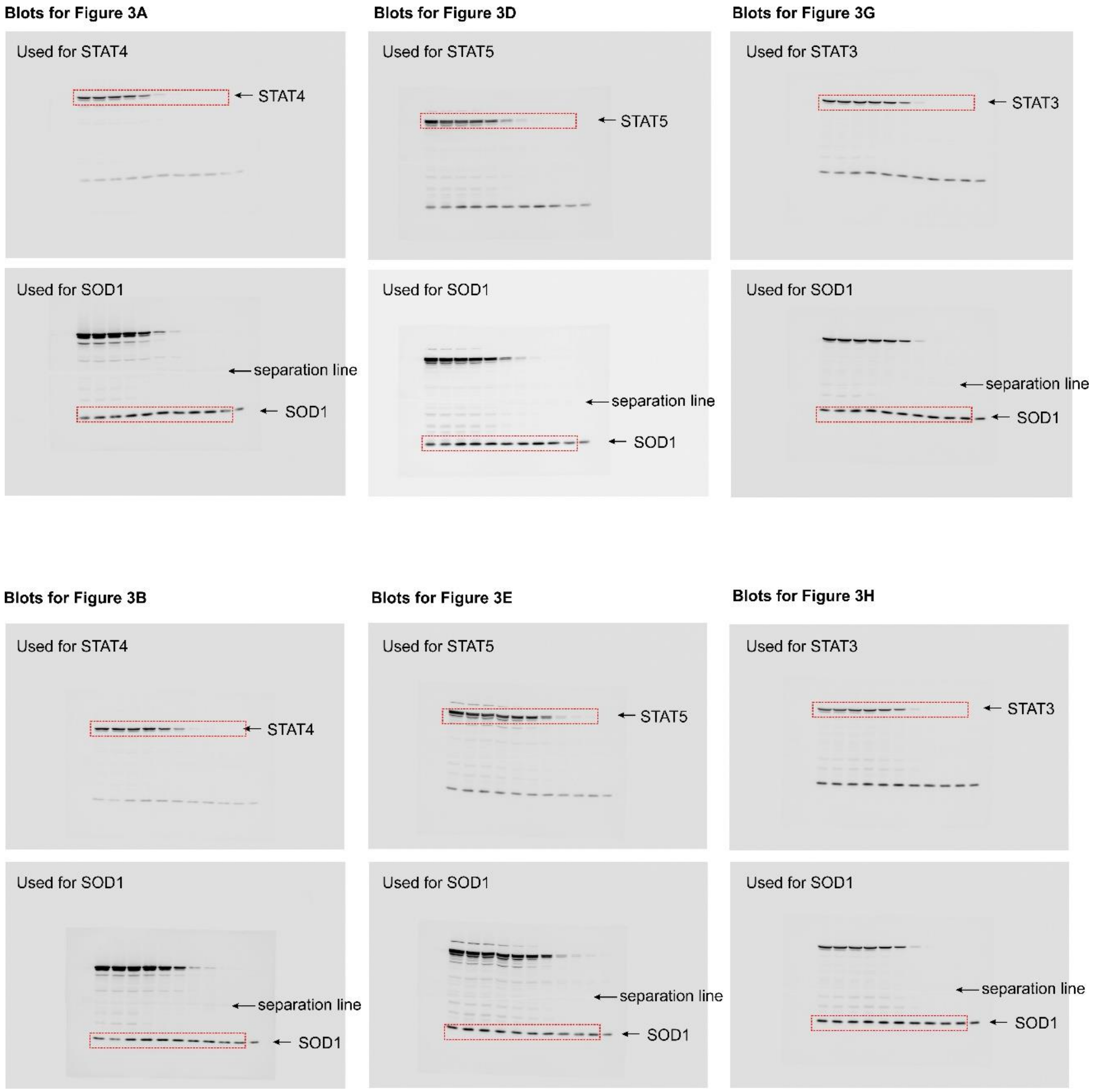

Figure S5: Uncropped pictures of data shown in Figure 3.

Figure S6

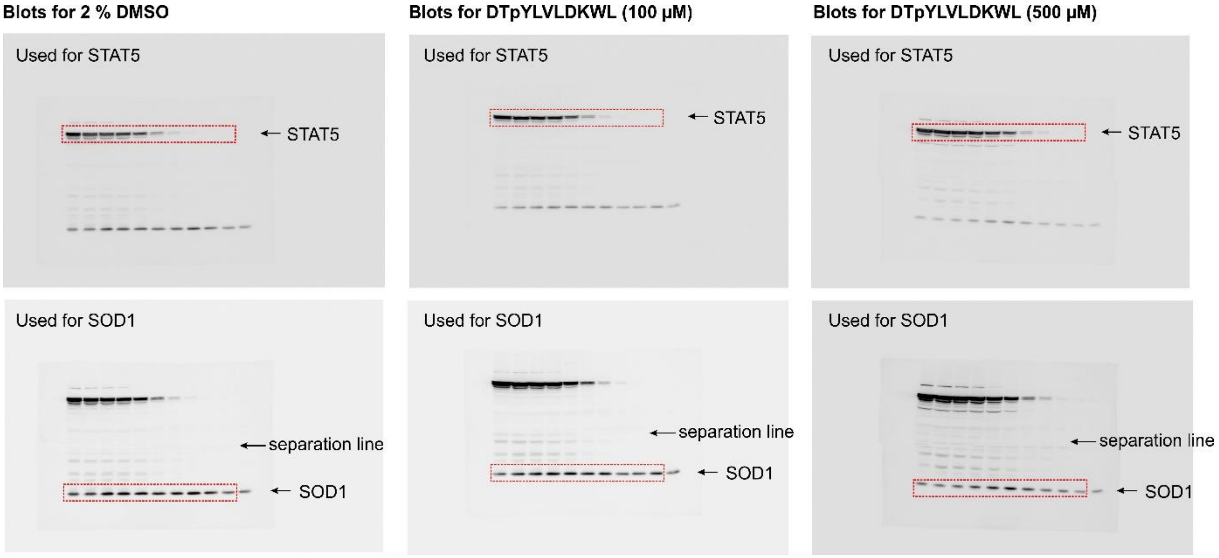

Figure S6: Uncropped pictures of data shown in Figure S4.

Table S1

Time	IC_{50} (µM) no reducing agent	IC_{50} (µM) 1 mM DTT	IC_{50} (µM) 1 mM TCEP
**1 hr**	2.2 ± 0.2	1.7 ± 0.3	2.4 ± 0.3
**2 hr**	2.4 ± 0.3	2.5 ± 0.4	2.4 ± 0.2
**3 hr**	2.6 ± 0.3	3.7 ± 0.6	2.5 ± 0.4
**4 hr**	2.4 ± 0.1	4.9 ± 0.9	2.8 ± 0.5

Table S1: Stability of STAT4 inhibition by PPADS over time with or without reducing agents. Mean values ± standard deviations are given (n = 3).

Table S2

Protein	Maximum HTRF ratio	Unlabeled peptide	IC_{50} unlabeled peptide (µM)
STAT3 (110 nM)	~8-25	Ac-pYLPQTV-NH_2	1.15 ± 0.14
STAT4 (30 nM)	~30-33	Ac-GpYLPQNID	2.01 ± 0.08
STAT5a (110 nM)	~24-34	DTpYLVLDKWL	2.48 ± 0.27
STAT5b (80 nM)	~27-47	DTpYLVLDKWL	4.18 ± 0.25

Table S2: Activity of inhibitory peptides against the respective STAT proteins in HTRF assays. The maximum HTRF ratio is the HTRF ratio obtained in the absence of inhibitor. Mean values ± standard deviations are given (n = 3).

Screening of chemical libraries

Screening of chemical libraries comprising approximately 5000 known bioactive compounds in fluorescence polarization-based assays against phosphorylation-dependent protein-protein interaction domains, including STAT proteins, has been described.[1] PPADS was purchased from Sigma-Aldrich, and iso-PPADS from Bio-Techne.

Fluorescence polarization assays

Competitive fluorescence polarization (FP) assays for STAT1,[2] STAT3,[3] STAT4,[4] STAT5a,[5] STAT5b[6] and STAT6[1] were performed as previously described. Briefly, peptide binding to proteins was analyzed using the following carboxyfluorescein (CF)-labelled peptides at a concentration of 10 nM: STAT1: 5-CF-GpYDKPHVL-OH, STAT3: 5-CF-GpYLPQTV-NH_2, STAT4: 5-CF-GpYLPQNID-OH, STAT5a and STAT5b: 5-CF-GpYLVLDKW-OH, STAT6: 5-CFGpYVPWQDLI-OH. Proteins were used at a final concentration representing the approximate K_d value for binding of the corresponding peptide to the protein, namely 65 nM for STAT1, 65 nM for STAT3, 35 nM for STAT4, 125 nM for STAT5a, 65 nM for STAT5b and 75 nM for STAT6. Assays were carried out in buffer containing 10 mM Tris/HCl, 50 mM NaCl, 1 mM EDTA, 0.1% NP-40 substitute and 2% DMSO, with 1 mM reducing agent where applicable, at pH 8.0. Proteins were incubated with compound for 1 h at room temperature before adding peptide and incubating for a further 1 h. FP was measured using an Infinite F500 plate reader (Tecan) and measurements were converted into percent inhibition according to the binding curve equation. Curves were plotted and best fits calculated using OriginPro software (OriginLab).

HTRF assays

Homogenous time-resolved fluorescence (HTRF) assays for STAT3, STAT4, STAT5a and STAT5b were carried out using Cisbio Monoclonal Antibody Anti-6His-Tb Gold as the fluorescence donor (Perkin Elmer) and the corresponding CF-labelled binding peptide as the acceptor. Protein at 110 nM (STAT3), 30 nM (STAT4), 110 nM (STAT 5a) or 80 nM (STAT5b) was combined with terbium-labelled antibody at the recommended concentration, 150 nM CF-labelled peptide and compound as indicated, and incubated at room temperature for 1 h. HTRF assays were carried out in buffer containing 10 mM Tris/HCl, 50 mM NaCl, 1 mM EDTA, 0.1% NP-40 substitute and 2% DMSO, without reducing agent. Fluorescence/luminescence was measured in Proxiplate 384-well plates (Perkin Elmer), using a 340 nm excitation filter with 520 nm and 620 nm emission filters, with 60 µs time delay. The average reading from 3 wells was obtained. After subtraction of the blank (buffer only) from each reading, the ratio of the 520 nm / 620 nm readings was calculated. The value of the 520 nm / 620 nm ratio in the absence of CF-peptide (negative control) was then subtracted from the ratio obtained in the presence of DMSO or test compound (dissolved in DMSO) at each concentration. The result of this

calculation was divided by the ratio (520 nm / 620 nm) of the negative control to give the HTRF ratio.[7] The maximum HTRF ratio is the HTRF ratio obtained in the absence of inhibitor and is defined as 100 % HTRF in the Figures.

Cell culture and lysates

NK-92 cells (DSMZ) were cultured in α-MEM containing 12.5% fetal bovine serum, 12.5% horse serum, 2 mM L-glutamine, penicillin/streptomycin antibiotics and 50 U/ml human recombinant IL-2 (all reagents from Gibco at Thermo Fisher). K562 cells (DSMZ) were cultured in RPMI 1640 medium containing 10% FBS, 2 mM L-glutamine and penicillin/streptomycin. For whole-cell lysates, cells were harvested by centrifugation, washed once with ice-cold PBS and resuspended at 3×10^6 cells per 100 µl in TBS supplemented with EDTA-free Halt Protease Inhibitor Cocktail (Fisher Scientific). Cells were lysed using three freeze-thaw cycles using liquid nitrogen and a room-temperature water bath. Lysates were cleared by centrifugation, aliquoted and frozen in liquid nitrogen before storage at -80°C.

CETSA

CETSA experiments were performed based on the published protocol.[8] Cell lysates were incubated with test compound or DMSO for 15 minutes at room temperature before heating in 50 µl aliquots to the designated temperatures in a Veriti Pro Thermocycler (Thermo Fisher) for 3 min, followed by 3 min at room temperature before placing ice. The samples were transferred to 1.5 ml tubes and protein aggregates removed by centrifugation at 20,000 g for 20 minutes at 4°C. The soluble fractions were analyzed by Western blotting.

Western blotting

Treated lysates were separated by SDS-PAGE on a 10% gel and transferred to nitrocellulose membrane using a TransBlot semi-dry blotting protocol (Bio-Rad Laboratories). Proteins of interest were detected using monoclonal rabbit primary antibodies (Cell Signaling), anti-rabbit-HRP-secondary antibody (Dako) and Pierce ECL Plus chemiluminescence reagent (Thermo Fisher). Bands were visualized using an ImageQuant system (GE Healthcare) and quantitated with ImageJ software (NIH).[9]

Supporting references

[1] M. Gräber, W. Janczyk, B. Sperl, N. Elumalai, C. Kozany, F. Hausch, T. A. Holak, T. Berg "Selective targeting of disease-relevant protein binding domains by O-phosphorylated natural product derivatives", *ACS Chem. Biol.* **2011**, *6*, 1008-1014.

[2] J. Schust, B. Sperl, A. Hollis, T. U. Mayer, T. Berg "Stattic: a small-molecule inhibitor of STAT3 activation and dimerization", *Chem. Biol.* **2006**, *13*, 1235-1242.

[3] J. Schust, T. Berg "A high-throughput fluorescence polarization assay for signal transducer and activator of transcription 3", *Anal. Biochem.* **2004**, *330*, 114-118.

[4] A. Berg, M. Gräber, S. Schmutzler, R. Hoffmann, T. Berg "A High-Throughput Fluorescence Polarization-Based Assay for the SH2 Domain of STAT4", *Methods Protoc.* **2022**, *5*, 93.

[5] N. Elumalai, A. Berg, K. Natarajan, A. Scharow, T. Berg "Nanomolar Inhibitors of the Transcription Factor STAT5b with High Selectivity over STAT5a", *Angew. Chem. Int. Ed.* **2015**, *54*, 4758-4763.

[6] J. Müller, J. Schust, T. Berg "A high-throughput assay for signal transducer and activator of transcription 5b based on fluorescence polarization", *Anal. Biochem.* **2008**, *375*, 249-254.

[7] J.-P. Leyris, T. Roux, E. Trinquet, P. Verdié, J.-A. Fehrentz, N. Oueslati, S. Douzon, E. Bourrier, L. Lamarque, D. Gagne, J.-C. Galleyrand, C. M’kadmi, J. Martinez, S. Mary, J.-L. Banères, J. Marie "Homogeneous time-resolved fluorescence-based assay to screen for ligands targeting the growth hormone secretagogue receptor type 1a", *Analytical Biochemistry* **2011**, *408*, 253-262.

[8] N. Brovchenko, A. Berg, S. Schubert, J. Grab, T. Munzel, C. Protzel, K. Natarajan, T. Berg "Biaryl Phosphates and Phosphonates as Selective Inhibitors of the Transcription Factor STAT4", *Angew. Chem. Int. Ed.* **2025**, *64*, e202504420.

[9] C. A. Schneider, W. S. Rasband, K. W. Eliceiri "NIH Image to ImageJ: 25 years of image analysis", *Nat. Methods* **2012**, *9*, 671-675.
